# Independent and additive contribution of white matter hyperintensities and Alzheimer’s disease pathology to basal forebrain cholinergic system degeneration

**DOI:** 10.1016/j.nicl.2023.103477

**Published:** 2023-07-17

**Authors:** Christine Kindler, Neeraj Upadhyay, Zeynep Bendella, Franziska Dorn, Vera C. Keil, Gabor C. Petzold

**Affiliations:** a Division of Vascular Neurology, Department of Neurology, University Hospital Bonn, Venusberg-Campus 1, 53127 Bonn, Germany; b German Center for Neurodegenerative Diseases (DZNE), Venusberg-Campus 1, 53127 Bonn, Germany; c Clinical Functional Imaging Group, Department of Diagnostic and Interventional Radiology, University Hospital Bonn, Venusberg-Campus 1, 53127 Bonn, Germany; d Department of Neuroradiology, University Hospital Bonn, Venusberg-Campus 1, 53127 Bonn, Germany; e Department of Radiology and Nuclear Medicine, Amsterdam UMC, VUmc, Amsterdam, The Netherlands; f Amsterdam Neuroscience, Amsterdam UMC, Amsterdam, The Netherlands

**Keywords:** Alzheimer's disease, Cerebral small-vessel disease, Cholinergic basal forebrain nucleus, Nucleus basalis of Meynert, White matter hyperintensities, Voxel-based morphometry

## Abstract

**Objectives:**

Degeneration of the cholinergic basal forebrain nuclei (CBFN) system has been studied extensively in Alzheimer's disease (AD). White matter hyperintensities are a hallmark of aging as well as a common co-morbidity of AD, but their contribution to CBFN degeneration has remained unclear. Therefore, we explored the influence of white matter hyperintensities within cholinergic subcortical-cortical projection pathways on CBFN volumes and regional gray matter volumes in AD and age- and gender-matched controls.

**Methods:**

We analyzed magnetic resonance images (MRI) from 42 patients with AD and 87 age- and gender-matched control subjects. We assessed the white matter hyperintensity burden within the cholinergic projection pathways using the Cholinergic Pathways Hyperintensities Scale (CHIPS), and applied probabilistic anatomical maps for the analysis of CBFN volumes, i.e. the Ch1-3 compartment and the Ch4 cell group (nucleus basalis of Meynert), by diffeomorphic anatomical registration using exponentiated lie algebra analysis of voxel-based morphometry. Using multiple linear regression analyses, we explored correlations between regional gray matter volumes and the extent of white matter hyperintensities or CBFN volumes in both groups.

**Results:**

In AD, all CBFN volumes were significantly smaller than in controls, and white matter hyperintensity burden within the cholinergic projection pathways was not correlated with CBFN volume. In controls, white matter hyperintensity burden within the cholinergic projection pathways was inversely correlated with CBFN volume when corrected for sex and total intracranial volume, but this correlation was no longer significant after correction for age. Voxel-wise multiple linear regression analyses using threshold-free cluster enhancement revealed that in controls, cholinergic pathway hyperintensities correlated with gray matter loss in perisylvian areas, whereas the were no effects in AD. Moreover, we found that CBFN volumes correlated with distinct regional cortical atrophy patterns in both groups.

**Conclusion:**

Our results indicate that white matter hyperintensities and AD pathology contribute independently but additively to the degeneration of cholinergic basal forebrain structures. Whereas AD is primarily associated with CBFN volume loss, cholinergic degeneration associated with white matter hyperintensities appears to involve disruption of cholinergic cortical projection fibers with less pronounced effects on CBFN volumes.

## Highlights

• Cholinergic basal forebrain nuclei (CBFN) are smaller in AD compared to controls.
• White matter hyperintensities in cholinergic projections are not correlated with CBFN volume in AD.
• Cholinergic pathway hyperintensities are only weakly correlated with CBFN volume in non-AD subjects.
• Cholinergic pathway hyperintensities correlate with gray matter atrophy in controls, but not in AD.
• CBFN volumes correlate with distinct regional cortical atrophy patterns in both groups.

## 1 Introduction

White matter hyperintensities (WMHs) on cerebral magnetic resonance imaging (MRI) are a hallmark of cerebral small-vessel disease and very common in the elderly population (Gouw et al., 2011, Wardlaw et al., 2015). They are highly variable in extent and location, ranging from small punctuate lesions to patchy or extensive confluent lesions in the deep or periventricular white matter (Cannistraro et al., 2019). Neuropathologically, WMHs are characterized by ischemic white matter changes, including demyelination, edema, gliosis, and axonal loss (Fazekas et al., 1993). Prevalence and severity of WMHs increase with age (Leeuw et al., 2001); moreover, they are associated with cardiovascular risk factors and clinically symptomatic cerebrovascular disease (Launer, 2004, Murray et al., 2005). WMHs can remain clinically silent, but may also correlate with gait disturbance, cognitive impairment, and an increased risk for stroke (Barber et al., 1999, Debette and Markus, 2010). Importantly, WMHs are also highly prevalent in Alzheimer's disease (AD) (Barber et al., 1999), but it has remained incompletely understood how they influence the expression and severity of AD-specific brain changes.

Several lines of evidence, including post-mortem histopathological studies as well as MRI-based studies, have reported that AD is associated with neurodegeneration and atrophy of cholinergic basal forebrain nuclei (CBFN). On the other hand, the evidence for an involvement of the cholinergic system in white matter pathology has remained less clear. Although some studies have reported pathological changes of the cholinergic forebrain system in the presence of WMHs (Gottfries et al., 1994, Hanyu et al., 2002, Hanyu et al., 2007, Kim et al., 2013, Liu et al., 2017, Nemy et al., 2020, Wallin et al., 1989), it has remained undetermined if these changes, similar to AD, are due to a primary neuropathology within the CBFN, or if WMHs affect the basal forebrain cholinergic system primarily by disrupting cholinergic white matter projection pathways, potentially resulting in Wallerian degeneration of the CBFN. Importantly, the latter case would argue for two separate, and perhaps additive, routes of pathological damage in patients with AD and WMHs. One quantifiable marker of these potentially separate routes of damage are cortical atrophy patterns, which may differ in conditions directly affecting the CBFN, such as AD, or those primarily affecting white matter projection pathways, such as WMHs. However, only few studies have investigated the correlation between CBFN atrophy and regional cortical atrophy (Cantero et al., 2017, Grothe et al., 2010, Kilimann et al., 2017, Wolf et al., 2014). Hence, it has remained unclear whether disease-specific cortical atrophy patterns correlate with cholinergic degeneration in AD, and if they are influenced by the presence of WMHs.

The main objective of this pilot study was to explore the influence of WMHs on CBFN volumes in patients with AD and in age- and gender-matched control subjects. Moreover, we investigated white matter integrity within the cholinergic cortical projection pathways. In addition, we compared the topographic distribution of regional gray matter atrophy patterns between AD and controls. We also explored relationships between regional cerebral gray matter volume and the extent of WMHs within cholinergic projection pathways, and tested the hypothesis that cholinergic projection pathway hyperintensities and CBFN volumes are correlated.

## 2 Materials and methods

### 2.1 Subjects

The research ethics committee of University Hospital Bonn confirmed that no ethical approval and informed consent of the subjects were required because of the retrospective nature and anonymized design of the study, and as only clinical routine data were used. We retrospectively analyzed 3D magnetization-prepared T1-weighted magnetic resonance images (3D T1w MPRAGE) and T2-weighted fluid-attenuated inversion recovery (FLAIR) images from our hospital-based radiological and clinical database. The search period ranged from October 2010 to April 2018. Patient local case mix-adapted database search terms were “cerebral small vessel disease”, “Alzheimer’s disease”, “headache”, “dizziness”, “syncope”, “paresthesia”, “exclusion of microembolization” and “exclusion of metastases”. MRIs of patients with reported stroke, non-vascular intracranial lesions or other structural changes were excluded. 43 subjects fulfilled the criteria of probable AD with evidence of the AD pathophysiological process (i.e. they i) met the core clinical criteria for probable AD dementia and ii) were amyloid-positive based on CSF or PET studies, or showed disproportionate atrophy of the medial temporal lobe) according to National Institute on Aging and Alzheimer's Association (NIA-AA) diagnostic guidelines (McKhann et al., 2011). One subject in the AD group had an extremely high CHIPS score and was removed from the analysis as an outlier. For comparison, 87 age- and gender-matched subjects not fulfilling the NIA-AA criteria for AD were selected as controls. The MRIs of both groups were examined for the presence of WMHs as well as the STandards for ReportIng Vascular changes on nEuroimaging (STRIVE) criteria (Wardlaw et al., 2013).

### 2.2 Imaging

#### 2.2.1 Image acquisition

Brain MRIs for this retrospective analysis had been acquired on 1.5 Tesla and 3 Tesla scanners on a clinical whole-body MRI system (Achieva TX, Philips Healthcare, Best, The Netherlands) using an 8-channel head coil. For the analysis, morphological brain imaging included sagittal 3-dimensional (3D) T1 weighted magnetization-prepared rapid acquisition with gradient echo (3D MPRAGE), axial fluid attenuated inversion recovery (FLAIR), diffusion-weighted imaging (DWI), susceptibility-weighted imaging (SWI or T2 FFE), and T2 or quantitative T2-weighted imaging ((q)T2W) (SupplementaryTable S1*)*.

#### 2.2.2 Image processing

For the structural analysis of whole brain GM and the subsequent region of interest analysis of basal forebrain cholinergic cell groups, a voxel-based morphometry using the Diffeomorphic Anatomical Registration by the Exponentiated Lie Algebra (DARTEL) algorithm was performed (Ashburner, 2007). Specifically, the anonymized raw T1-weighted images were first converted from Digital Imaging and Communications in Medicine (DICOM) into Neuroimaging Informatics Technology Initiative (NIfTI) format using MRIcroGL software. Further image processing and analysis were performed using the Statistical Parametric Mapping software (SPM12) running on MatLab (R2021b, Mathworks). All 3D T1-weighted MRI scans were visually inspected for anatomical artifacts, motion artifacts and incomplete whole-brain coverage. Each image was then manually reoriented and the origin set to the anterior commissure at the level of the interhemispheric plane (Talairach and Tournoux, 1988).

Subsequently, images were segmented into gray matter (GM), white matter (WM) and cerebrospinal fluid (CSF) using the standard unified segmentation module (Ashburner and Friston, 2005). We used the DARTEL algorithm (Ashburner, 2007) to create a study-specific GM template for spatial normalization of the segmented images of each subject. This algorithm uses an alternating procedure between computing a group template and warping the individual tissue probability maps into alignment with this template to finally create an individual flow field of each participant (Ashburner, 2009, Ashburner and Friston, 2009). The GM template was normalized to MNI space and the resulting flow fields were applied to the individual GM segments of each participant. Voxel values were modulated to preserve the original amount of GM volume present for normalization. The resulting images were then spatially normalized in the MNI space, modulated, resliced (1.5-mm isotropic voxels) and smoothed with a 6-mm full-width at half maximum Gaussian kernel. Finally, all preprocessed GM maps were visually checked for segmentation and registration artifacts.

Next, the individual volumes of GM, WM and CSF were computed using the modulated images of the three tissue classes. The individual total intracranial volume (TIV) was calculated as the sum of all three tissue classes and used as covariate.

To perform a voxel gray matter evaluation of the CBFN cell groups, the right- and left hemispheric cholinergic cell groups Ch1-3 and Ch4 (nucleus basalis of Meynert) were identified separately in each individual subject using probabilistic anatomical maps in MNI space (Zaborszky et al., 2008). The maps are available in the SPM 12 Anatomy Toolbox (Eickhoff et al., 2005) and have been used in previous imaging studies (Lammers et al., 2018). The GM volumes were then calculated using the MATLAB get_totals script on the modulated, normalized and unsmoothed GM segmented images.

**Fig. 1:**
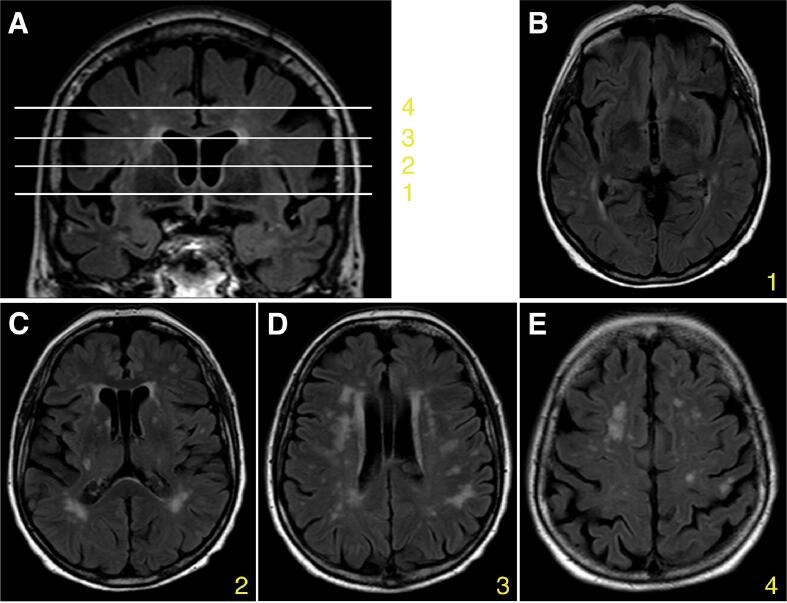
Anatomical levels assessed using the Cholinergic Pathways Hyperintensities (CHIPS) scale. (A) Representative example. White matter lesions within cholinergic projection pathways were assessed on four anatomical levels: 1, low external capsule; 2, high external capsule; 3, corona radiata; 4, centrum semiovale. (B-E) Axial planes of the anatomical levels depicted in the representative example in (A) that were used for the assessment of the CHIPS scores: 1, lower external capsule (CHIPS score: 16); 2, high external capsule (CHIPS score: 20); 3, corona radiata (CHIPS score: 8); 4, centrum semiovale (CHIPS score: 5).

FLAIR and T2-weighted axial sequence acquisitions were used to assess the presence of WMHs, lacunes and enlarged perivascular spaces by board-certified neuroradiologists. Perivascular spaces were classified in 4 groups: Type I along lentico-striatal arteries (LSAs) in the basal ganglia, type 2 along perforating medullary arteries in the white matter, type III along penetrating collicular arteries in the midbrain and thalami, and type IV in the opercula or anterior temporal lobe. Where available, additional coronal acquisitions were inspected for confirmation. A board-certified neuroradiologist also rated all cases according to the Cholinergic Pathways Hyperintensities Scale (Bocti et al., 2005). CHIPS has a range from 0 to 100 with stronger weighting of basal white matter hyperintensities assessed on four levels in the axial plane and further sets a severity level based on the affection of less or more than 50% of that region (Fig. 1). Diffusion-weighted images were used to detect recent small subcortical infarcts, and susceptibility-weighted images were used to detect cerebral microbleeds.

Temporal lobe atrophy was assessed on coronal T1-weighted images using standard procedures (Scheltens et al., 1992).

### 2.3 Statistical analysis

#### 2.3.1 Demographic and imaging data

Statistical analysis of demographic and imaging data was performed using the Statistical Package for the Social Sciences Statistics for Mac version 26 (SPSS, IBM. Armonk, NY, USA). For the demographic data and the volumes of the individual brain regions, normality of the distribution was tested using the Shapiro-Wilk test and equality of variance was tested with the Levene test.

The group comparison of not normally distributed or ordinal data was calculated with the Mann Whitney-*U* test; otherwise, the unpaired *T*-test was used. As the assumptions of the chi-square omnibus did not hold for gender, this variable was examined using Fisher’s exact test.

#### 2.3.2 Whole brain gray matter analysis

First, we investigated whole brain GM differences between AD and controls. To this end, voxel-wise whole brain comparisons were performed using the General Linear Model (GLM). We compared AD with controls and vice versa; age, gender and TIV served as nuisance covariates.

To explore the impact of WMHs within cholinergic pathways on regional gray matter volume, voxel-based whole-brain multiple linear regression analysis with the CHIPS scores as regressor was applied separately in AD and controls. Age, gender and TIV as nuisance covariates were entered into the design matrix. For each assay, TFCE (Threshold Free Cluster Enhancement) nonparametric tests were performed and each test were randomly permuted 5000 times (Smith and Nichols, 2009). The significance levels were set to p < 0.001. In case of missing significant results, significance levels were exploratively increased to p < 0.05. All results were corrected applying a family-wise error (FWE) correction for multiple comparisons.

#### 2.3.3 Analysis of CBFN volumes

First, we explored differences of CBFN volumes between AD and controls. To correct for individual brain size, the individual volumes of the right- and left hemispheric cholinergic cell groups were divided by the corresponding TIV. The resulting volumes were compared using unpaired t-tests. The level of statistical significance was set to *p* < 0.05. Cohen's *d* was calculated to determine the corresponding effect size.

To evaluate the impact of WMHs on the basal forebrain cholinergic system, we separately calculated correlations between CBFN volumes and CHIPS scores in controls and AD. Spearman's rank correlation was applied as a non-parametric approach. The correlation analysis was initially performed without corrections, and subsequently repeated several times, each time corrected for confounding factors (age, gender, and TIV).

To investigate whether the volumes of the left and right Ch4 (nucleus basalis of Meynert) correlate with loss of regional cerebral gray matter volume, voxel-based multiple linear regression analyses of the whole brain were performed for both sides separately. In each case, the respective volumes served as predictive regressor and age, sex, and TIV as nuisance covariates. Non-parametric TFCE tests were performed for each assay and each test was randomly permuted 5000 times (Smith and Nichols, 2009). Significance levels were set at *p* < 0.001, corrected for multiple comparisons using FWE.

## 3 Results

### 3.1 Demographic and imaging characteristics

42 subjects who met the criteria for probable AD and 87 gender- and age-matched subjects from our hospital-based radiological and clinical database were included in the analysis. One subject from the AD group showed a very high CHIPS score of 88 and therefore was considered as an outlier and excluded from the further analyses. Compared to the control group, AD was associated with lower gray matter volume and higher CSF volume (Table 1). No significant differences were found for age, gender, CHIPS score, white matter volume, TIV and the STRIVE criteria for cerebral small-vessel disease, including frequencies of recent small subcortical infarcts, lacunes, cerebral microbleeds and the presence of enlarged perivascular spaces. As expected, the extent of temporal lobe atrophy was significantly more pronounced in AD than in controls (Table 1).

**Table 1:** Demographic data and group statistics of AD and controls.

	**Controls**	**AD**	**Test**	**p value**
**n**	87	41		
**Age (years)**	71.94 ± 10.28	72.22 ± 7.33	T = −0.174 (df = 106.138)	0.862^a^
**Gender (women/men)**	47/40	21/20	exact χ^2^ = 0.307 (df = 1)	0.705^b^
**CHIPS score**	22.78 ± 18	22.17 ± 16.04	U = 1757.000	0.892^c^
**GM volume (ml)**	568.36 ± 69.36	505.86 ± 57.82	T = 5.006 (df = 126)	> 0.001^a^
**WM volume (ml)**	420.52 ± 61.65	403.05 ± 52.79	U = 1503.000	0.152^c^
**CSF volume (ml)**	410.46 ± 103.84	509.13 ± 85.26	T = −5.298 (df = 126)	> 0.001^a^
**TIV (ml)**	1399.33 ± 0146.06	1418.04 ± 0132.32	U = 1634.000	0.445^c^
**Recent small subcortical infarct (yes/no)**	52/1 (n = 53)	35/0 (n = 35)	exact χ^2^ = 0.668 (df = 1)	1.000^b^
**Lacunes (0/1**–**5/6**–**10/>10)**	44/35/1/7	17/17/5/2	exact χ^2^ = 13.577 (df = 8)	0.093^b^
**Perivascular space (I + II/III/IV)**	47/19/21	16/9/16	exact χ^2^ = 3.410 (df = 2)	0.182^b^
**Cerebral microbleeds (yes/no)**	3/10 (n = 13)	7/17 (n = 24)	exact χ^2^ = 0.159 (df = 1)	1.000^b^
**MTA score right**	1.15 ± 0.98	2.98 ± 0.90	U = 365.000	> 0.001^c^
**MTA score left**	1.17 ± 0.94	2.90 ± 0.98	U = 428.000	> 0.001^c^

Results for age, CHIPS score and total intracranial volume (TIV) are displayed as mean ± standard deviation. a, unpaired *t* test; b, Fisher’s exact test; c, Mann Whitney-*U* Test (U); CSF, cerebrospinal fluid; GM, gray matter; WM, white matter.

### 3.2 CBFN volumes are reduced in AD

**Fig. 2:**
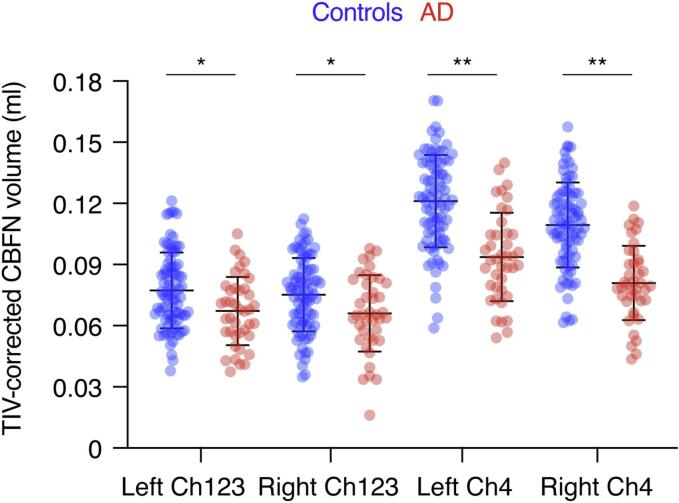
CBFN volumes are smaller in AD compared to controls. Cholinergic basal forebrain nuclei (Ch1-3, Ch4; corrected for total intracranial volume) were significantly smaller in AD compared to controls (unpaired *t*-test for each comparison; * *p* < 0.05; ** *p* < 0.001).

All cholinergic cell group volumes were significantly smaller in AD compared to controls (*p*_L_Ch123_ = 0.002; *p*_R_Ch123_ = 0.004; *p*_L_Ch4_ < 0.001; *p*_R_Ch4_ < 0.001; Fig. 2; Table S2), indicating, considering that small-vessel pathology markers were similarly expressed in both groups, that AD pathology has a stronger contribution to basal forebrain nuclei degeneration than small-vessel pathology. The most pronounced atrophies were found for the left and right nucleus basalis of Meynert (Ch4; *d*_L_Ch4_ = 1.228; *d*_R_Ch4_ = 1.417), whereas the effect size for the left and right medial septum and the diagonal band of Broca (Ch1-3) was moderate (*d*_L_Ch123_ = 0.563; *d*_R_Ch123_ = 0.504).

### 3.3 The effects of AD pathology and age on CBFN volumes predominate over those by cholinergic pathway hyperintensities

**Fig. 3:**
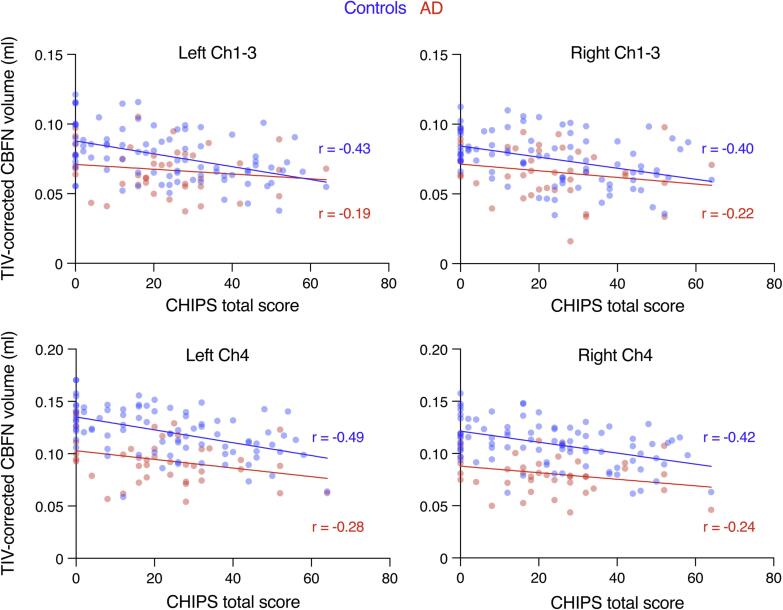
CBFN volumes and cholinergic pathway hyperintensities are only weakly correlated. Scatter plots with linear regression lines and Spearman r values showing the relationship between volumes of the left and right cholinergic cell groups (Ch1-3 and Ch4; corrected for total intracranial volume) and cholinergic pathway hyperintensities (CHIPS scores) in AD and controls.

Next, we evaluated the impact of the presence of WMHs within the basal forebrain cholinergic system, quantified by the CHIPS score, in AD and controls. In control subjects, correlation analyses revealed moderate negative associations between CHIPS scores and CBFN volumes (Table 2), which remained stable after correction for gender and TIV, but lost significance after correction for age (Fig. 3 and Table 2). In AD, CHIPS scores and CBFN volumes were negatively correlated as well, but this correlation did not reach significance before or after correction, and effect sizes were generally small (Fig. 3 and Table 3).

**Table 2:** Correlation between CBFN volumes and cholinergic pathway hyperintensities in control subjects.

	**Uncontrolled**		**Age (df = 84)**		**Gender (df = 84)**		**TIV (df = 84)**	
	***r***	***p***	***r***	***p***	***r***	***p***	***r***	***p***
**left Ch1-3**	−0.433	< 0.001**	−0.175	0.108	−0.442	< 0.001**	−0.418	< 0.001**
**right Ch1-3**	−0.397	< 0.001**	−0.103	0.347	−0.404	< 0.001**	−0.383	< 0.001**
**left Ch4**	−0.492	< 0.001**	−0.179	0.098	−0.511	< 0.001**	−0.482	< 0.001**
**right Ch4**	−0.420	< 0.001**	−0.110	0.313	−0.440	< 0.001**	−0.407	< 0.001**

Spearman correlation analyses with correlation coefficient *r* and *p* values (uncontrolled and after correction for age, gender and total intracranial volume (TIV), respectively).

**Table 3:** Correlation between CBFN volumes and cholinergic pathway hyperintensities in AD.

	**Uncontrolled**		**Age (df = 38)**		**Gender (df = 38)**		**TIV (df = 38)**	
	***r***	***p***	***r***	***p***	***r***	***p***	***r***	***p***
**left Ch1-3**	−0.191	0.231	−0.090	0.580	−0.173	0.287	−0.205	0.205
**right Ch1-3**	−0.224	0.159	−0.106	0.514	−0.205	0.204	−0.242	0.133
**left Ch4**	−0.283	0.073	−0.214	0.184	−0.270	0.092	−0.303	0.057
**right Ch4**	−0.240	0.130	−0.148	0.361	−0.234	0.146	−0.254	0.113

Spearman correlation analyses with correlation coefficient *r* and *p* values (uncontrolled and after correction for age, gender and total intracranial volume (TIV), respectively).

These data indicate that potential effects of WMHs within cholinergic pathways on CBFN volumes are small and appear to be dominated by effects of age or AD pathology.

### 3.4 The impact of WMHs on cortical atrophy patterns is dominated by effects of concomitant AD pathology

**Fig. 4:**
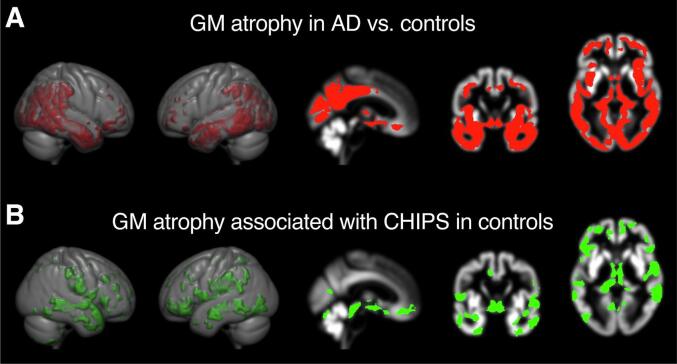
Specific regional cortical atrophy patterns in AD and controls. Regional gray matter (GM) volume loss represented by TFCE maps were superimposed on a gray matter skull averaged over all subjects (TFCE; 5000 permutations; FWE-corrected). A gray matter skull as well as axial, coronal, and sagittal sections are shown. The right side of the axial images corresponds to the right side of the natural brain. (A) Regional GM atrophy patterns in AD patients compared to controls (significant reductions are shown in red). (B) Regional GM atrophy patterns (green) associated with WMH burden in cholinergic pathways (CHIPS scores) in the control group. (For interpretation of the references to colour in this figure legend, the reader is referred to the web version of this article.)

Next, we investigated differences in cortical atrophy patterns between AD and controls. Compared to controls, AD showed distinct cortical gray matter atrophy patterns bilaterally in medial and lateral temporal cortex, extending into the adjacent parietal and occipital and frontal cortical areas. As expected for AD (Whitwell et al., 2007), atrophies were most pronounced in the medial temporal lobe. Furthermore, we found a significant reduction of gray matter volume in the bilateral thalamus, cingulate gyrus and the basal forebrain compared to controls (Fig. 4A and Supplementary Table S3).

Using voxelwise multiple linear regression analyses, we subsequently examined the effects of WMHs within cholinergic subcortical-cortical projection pathways, quantified by the CHIPS score, on regional GM in both groups. At a threshold of p < 0.001, the CHIPS score correlated with atrophy in one cluster located in the right temporal pole in controls, while no significant correlations were found in AD (Supplementary Table S4). At a less stringent threshold of *p* < 0.05, we found a strong correlation between the CHIPS score and perisylvian areas extending to widespread fronto-parieto-temporal regions as well as the right sulcus calcarinus in controls (Fig. 4B, Supplementary Table S5). In contrast, even at that threshold, no cortical atrophy pattern correlating with the CHIPS score was observed in AD.

These data suggest that WMHs are associated with distinct cortical atrophy patterns, but that in AD these effects are dominated by the effects of AD pathology.

### 3.5 Specific regional cortical atrophy patterns related to Ch4 volume loss in the context of controls and AD

Next, using voxel-wise multiple linear regression analyses, we investigated whether CBFN volume reductions were associated with specific regional GM volume reduction patterns in both groups. Because our data had revealed that Ch4 (nucleus basalis of Meynert) had shown the largest volume loss in the AD group compared to controls, we focused on this nucleus for our analysis.

**Fig. 5:**
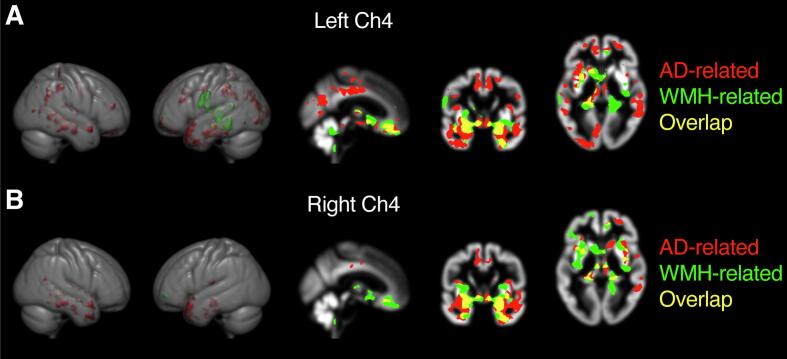
Specific regional cortical atrophy patterns related to Ch4 volume loss in controls and AD. Regional gray matter loss related to volume reduction of the (A) left and (B) right Ch4 (nucleus basalis of Meynert) represented by TFCE maps and superimposed on a gray matter skull averaged over all subjects (TFCE; 5000 permutations; FWE corrected; *p* < 0.001). A gray matter skull as well as axial, coronal, and sagittal sections are shown. The right side of the axial images correspond to the right side of the natural brain. Atrophic areas are depicted in red in the AD group and in green in the control group; overlapping areas are depicted in yellow. (For interpretation of the references to colour in this figure legend, the reader is referred to the web version of this article.)

We found that Ch4 volume was correlated with widely distributed extensive bilateral areas in all lobes in AD (Fig. 5; Supplementary Tables S6-S7), whereas in controls the associated cortical atrophies were much less widespread and affected mainly frontal, temporal, and cerebellar areas (Fig. 5; Supplementary Tables S8-S9).

## 4 Discussion

Several lines of evidence suggest that AD and small-vessel pathology can both lead to dysfunction of the basal forebrain cholinergic system (Grothe et al., 2010, Hanyu et al., 2002, Liu et al., 2017, Nemy et al., 2020). Yet, whether cholinergic dysfunction in AD is mechanistically related to separate pathogenic pathways – damage of white matter cholinergic projection pathways induced by WMHs, and primary degeneration of cholinergic nuclei in the basal forebrain – has remained incompletely understood. Comparing a cohort of AD patients with a cohort matched for age, gender and the presence of WMHs, we here found that AD is associated with stronger CBFN atrophy, particularly in the Ch4 subnucleus. Our data on AD are in line with previous histopathological studies examining the CBFN, which revealed a reduced cell count, although with large variations between individual subjects (Arendt et al., 1985, Cullen et al., 1997, Jung et al., 2012, Mufson et al., 2000, Whitehouse et al., 1981). Moreover, more recent MRI-based studies reported atrophies of the CBFN associated with AD, and in particular early and marked degeneration of Ch4 (Grothe et al., 2010, Grothe et al., 2012, Kilimann et al., 2014). Furthermore, we also found relationships between atrophy of individual CBFNs and age, which is also consistent with previous studies (Grothe et al., 2012, Grothe et al., 2013, Hanyu et al., 2002, Mann et al., 1984, Wolf et al., 2014) and may contribute to cholinergic deficits in old age independent of concomitant neurodegeneration.

Moreover, we found that the relationship between the extent of WMHs in white matter cholinergic projection pathways and CBFN atrophy was dominated by effects of age or AD pathology. In addition, the extent of CBFN volume loss was associated with specific cortical atrophy patterns that were distinct between AD and controls.

Thus, our data indicate that cholinergic dysfunction in AD is primarily driven by degeneration of basal forebrain nuclei, in particular the basal nucleus of Meynert, whereas cholinergic deficits in elderly non-AD subjects may be mostly driven by the disruption of cholinergic projection fibers caused by strategically located WMHs, potentially inducing retrograde axonal (Wallerian) degeneration. This potential effect of small-vessel pathology in the white matter on cholinergic projection fibers is supported by histopathological studies showing reduced cholinergic fiber density in cerebral small-vessel disease (Jung et al., 2012, Tomimoto et al., 2005).

Moreover, our data suggest that in the presence of AD pathology, the effect sizes for additional effects of WMHs within cholinergic projections pathways on CBFN volume loss are small, indicating that they are dominated by direct effects of AD.

Interestingly, we also found that regional cortical atrophy patterns correlated with the extent of WMHs within the cholinergic pathways in controls, but not in AD. Perisylvian regions and widely distributed fronto-parietotemporal cortical areas showed the strongest correlations in non-AD subjects. Such regional atrophies of perisylvian regions have been described in cerebral small-vessel disease (Habes et al., 2016, Kim et al., 2012, Seo et al., 2010, Seo et al., 2012). After passing the anterior cap, the cholinergic projection pathways spread into the lateral perisylvian division, connected to the opercula and insular cortex (Selden et al., 1998), perhaps explaining the anatomical connection between cholinergic projection pathway affection by WMHs and cortical degeneration of perisylvian areas. Interestingly, we did not find any regional cortical atrophy patterns correlating with the extent of WMHs within cholinergic pathways with our prespecified thresholds in AD. Although the reasons for these differences remain to be explored, a possible explanation would be that WMH-related changes in cortical atrophy patterns are overridden by mechanisms specific to AD pathophysiology, such as amyloid and tau deposition.

Finally, we also found that cortical atrophy patterns correlated with CBFN volumes in the basal forebrain. These patterns were disease-specific, in that extensively distributed areas in all lobes were associated with Ch4 volume in AD, but only small effects of Ch4 volume on cortical atrophy patterns were found in control subjects. This may indicate that the network effects of primary degeneration of Ch4 are primarily relevant and widespread in AD, but comparatively small in controls. The correlation between Ch4 volume and cortical atrophy in AD corroborates previous histopathological (Cullen et al., 1997) and MRI-based studies (Teipel et al., 2005). However, to our knowledge, our study is the first to indicate that WMHs appear to only play a minor role in these changes.

### 4.1 Limitations

Our pilot study has the typical limitations of a retrospective cross-sectional study in a clinical setting. First, subjects were measured in different scanners with different field strengths as well as different sequence parameters, which may have induced additional variance in the volumetric measurements. Second, although we selected the control group for the absence of established and sensitive criteria of AD, we cannot exclude that subjects in the control group had clinically silent AD-related pathology. Moreover, because of the retrospective nature using routine clinical data, neuropsychological assessments were not available. In addition, CBFN volumes and the WMHs are both highly colinear with age, and hence effects attributed to age may also be explained by these variables. Third, samples sizes were relatively low.

Future biomarker-based studies with multimodal imaging and neuropsychological test batteries are needed to more precisely determine the extent to which morphological changes in cholinergic nuclei and pathways are specific for the distinct pathologies and affect regional cortical brain structures and cognitive functions. Moreover, prospective studies using unified imaging protocols may also enable the sub-stratification using small-vessel markers other than WMHs, such as cerebral microbleeds.

### 4.2 Conclusion

Our results indicate that regional cortical atrophies correlated with cholinergic dysfunction are primarily related to CBFN (in particular Ch4) atrophy in AD, but that they are primarily related to disruption of cholinergic cortical projection fibers, with relative preservation of the CBFN, in the context of WMHs in non-AD subjects. Hence, our results support the notion that AD and small-vessel pathology contribute additively, and through distinct mechanisms, to cholinergic and cortical degeneration.

### CRediT authorship contribution statement

**Christine Kindler:** Conceptualization, Methodology, Formal analysis, Investigation, Writing – original draft, Visualization. **Neeraj Upadhyay:** Methodology, Formal analysis. **Zeynep Bendella:** Investigation, Resources. **Franziska Dorn:** Resources. **Vera C. Keil:** Formal analysis, Investigation, Resources. **Gabor C. Petzold:** Writing – original draft, Writing – review & editing, Visualization, Supervision, Funding acquisition.

## Declaration of Competing Interest

The authors declare that they have no known competing financial interests or personal relationships that could have appeared to influence the work reported in this paper.

## Data Availability

Data will be made available on request.
