# Supplementary material for: Independent and additive contribution of white matter hyperintensities and Alzheimer’s disease pathology to basal forebrain cholinergic system degeneration
Source: Neuroimage Clin. 2023 Jul 17;39:103477. doi: 10.1016/j.nicl.2023.103477 (PMC10387606; doi:10.1016/j.nicl.2023.103477)
Supplement: Supplementary data 1 [file mmc1.docx]

**Supplementary Material**

***Table S1.***

| **Magnetization prepared sagittal rapid gradient echo T1-weighted magnetic resonance images** | | | | | | |
| --- | --- | --- | --- | --- | --- | --- |
| **Scanner** | **Field strength** | **Sequence** | **Orientation** | **Voxel size (mm)** | **TE (ms)** | **TR (ms)** |
| Intera | 1.5 Tesla | 3D T1 | sagittal | 1x1x1 | 3.6 | 15.4 |
|  |  |  |  |  | 3.85 | 8.37 |
|  |  |  |  |  | 3.6 | 15.61 |
|  |  |  |  |  |  | 15.64 |
| Intera | 3 Tesla |  |  |  | 3.68 | 8.05 |
| Achieva |  |  |  |  | 3.5 | 7.56 |
| Ingenia |  |  |  |  | 3.19 | 7.06 |
|  | | | | | | |
| **Fluid-attenuated inversion recovery images (T2-FLAIR)** | | | | | | |
| **Scanner** | **Field strength** | **Sequence** | **Orientation** | **Voxel size (mm)** | **TE (ms)** | **TR (s)** |
| Achieva | 3 Tesla | 2D FLAIR | axial | 1x1x5 | 140 | 12 |
| Intera |  |  |  | 1x1x6 |  |  |
| Intera | 1.5 Tesla |  |  | 1.1x1.1x6 | 120 | 6 |
|  | | | | | | |
| **T2 –weighted axial sequence** | | | | | | |
| **Scanner** | **Field strength** | **Sequence** | **Orientation** | **Voxel size (mm)** | **TE (ms)** | **TR (s)** |
| Achieva | 3 Tesla | 2D-TSE | axial | 1x1x5 | 80 | 4.0 |
| Intera |  |  |  | 1x1x6 |  | 3.27 |
| Intera | 1.5 Tesla |  |  | 1.1x1.1x6 | 100 | 4.87 |
| **DWI –weighted axial sequence** | | | | | | |
| **Scanner** | **Field strength** | **Sequence** | **Orientation** | **Voxel size (mm)** | **TE (ms)** | **TR (s)** |
| Achieva | 3 Tesla | 2D-DWI | axial | 1x1x5 | 41 | 2725 |
| Intera |  |  |  | 1x1x6 |  |  |
| Intera | 1.5 Tesla |  |  | 0.98x0.98x5 | 78 | 2921 |
| **susceptibility –weighted axial sequence** | | | | | | |
| **Scanner** | **Field strength** | **Sequence** | **Orientation** | **Voxel size (mm)** | **TE (ms)** | **TR (s)** |
| Achieva | 3 Tesla | 3D-SWI or 2D T2W FFE | axial | 0.6x0.6x2 | 0 | 31 |
| Intera |  |  |  | 1x1x6 |  |  |
| Intera | 1.5 Tesla |  |  | 0.9x0.9x6 | 23 | 764 |

FLAIR: fluid-attenuated inversion recovery; TE: echo time; TR: repetition time; TSE: Turbo Spin-Echo.

***Table S2. CBFN volumes in AD and controls.***

|  | **Controls** | **AD** | **Test statistics^a^** | ***p* value** | ***d*** |
| --- | --- | --- | --- | --- | --- |
| **n** | 87 | 42 |  |  |  |
| **left Ch123** | 0.0774 ± 0.0186 | 0.0672 ± 0.0168 | T(126) = 2.974 | 0.002* | 0.563 |
| **right Ch123** | 0.0753 ± 0.0180 | 0.0661 ± 0.0188 | T(126) = 2.661 | 0.004* | 0.504 |
| **left Ch4** | 0.1211 ± 0.0226 | 0.0937 ± 0.0217 | T(126) = 6.481 | <0.001** | 1.228 |
| **right Ch4** | 0.1094 ± 0.0209 | 0.0810 ± 0.0182 | T(126) = 7.479 | <0.001** | 1.417 |

Mean values and standard deviations of the brain size corrected left and right cholinergic cell group volumes of 1-3 and 4 for CSVD and the mixed AD/CSVD respectively in milliliter. Group differences were assessed by an unpaired t test. To evaluate the corresponding effect size Cohen’s *d* was calculated.

***Table S3. Gray matter volume loss in AD compared to controls.***

| **Anatomical region** | **BA** | **Cluster size** | **TFCE** | **MNI coordinates** | | |
| --- | --- | --- | --- | --- | --- | --- |
|  |  |  |  | **x** | **y** | **z** |
| **R hippocampus** |  | **531958** | **72458.52** | **36** | **-27** | **-9** |
| R hippocampus |  |  | 70906.88 | 28 | -30 | -7 |
| R amygdala |  |  | 70905.45 | 21 | 0 | -15 |
| **R inferior frontal gyrus, triangular part** |  | **7** | **20706.60** | **47** | **27** | **12** |
| **L inferior temporal gyrus** |  | **1** | **18038.15** | **-55** | **-29** | **-30** |
| **L medial orbital gyrus** | **11** | **8** | **16760.40** | **-11** | **16** | **-24** |
| **L medial orbital gyrus** |  | **1** | **15522.65** | **-16** | **21** | **-27** |
| **R parietal operculum** | **40** | **1** | **15522.65** | **55** | **-24** | **16** |
| **L postcentral gyrus** | **4** | **1** | **15522.65** | **-29** | **-33** | **54** |
| **L cerebellum, lobule VIII** |  | **1892** | **15050.49** | **-21** | **-69** | **-40** |
| L cerebellum, crus II |  |  | 14903.57 | -14 | -78 | -39 |
| L cerebellum, lobule VIII |  |  | 14897.42 | -12 | -70 | -36 |

Cluster size = number of voxels in each significant cluster; TFCE = threshold free cluster enhancement, represents a statistical value, which combines voxel-height and cluster size; MNI coordinates (mm) = location of the maximum intensity voxel, given as spatial coordinate values in standard Montreal Neurological Institute coordinate space (mm). All the areas were significant at *p*<0.001, FWE corrected at a TFCE-based result.

***Table S4. Gray matter volume loss associated with CHIPS score in controls.***

| **Anatomical region** | **BA** | **Cluster size** | **TFCE** | **MNI coordinates** | | |
| --- | --- | --- | --- | --- | --- | --- |
|  |  |  |  | **x** | **y** | **z** |
| **R temporal pole** | **38** | **209** | **5515.66** | **44** | **14** | **-25** |

Cluster size = number of voxels in each significant cluster; TFCE = threshold free cluster enhancement, represents a statistical value, which combines voxel-height and cluster size; MNI coordinates (mm) = location of the maximum intensity voxel, given as spatial coordinate values in standard Montreal Neurological Institute coordinate space (mm). All the areas were significant at *p*<0.001, FWE corrected at a TFCE-based result.

***Table S5. Gray matter volume loss associated with CHIPS score in controls.***

| **Anatomical region** | **BA** | **Cluster size** | **TFCE** | **MNI coordinates** | | |
| --- | --- | --- | --- | --- | --- | --- |
|  |  |  |  | **x** | **y** | **z** |
| **R temporal pole** | **38** | **195508** | **5515.66** | **44** | **14** | **-25** |
| L basal forebrain | 34 |  | 5063.44 | -10 | 4 | -15 |
| L basal forebrain | 34 |  | 4948.18 | -24 | 6 | -17 |
| **L cerebellum** |  | **151** | **3985.08** | **-20** | **-37** | **-42** |
| **R anterior cingulate gyrus** | **32** | **2584** | **3488.65** | **10** | **31** | **30** |
| R anterior cingulate gyrus | 32 |  | 3455.65 | 15 | 35 | 24 |
| R anterior cingulate gyrus | 32 |  | 3418.02 | 14 | 22 | 34 |
| **L anterior cingulate gyrus** | **10** | **87** | **3386.58** | **-2** | **51** | **9** |
| **R hippocampus** |  | **130** | **3279.38** | **33** | **-8** | **-19** |
| **R lateral orbital gyrus** | **47** | **9** | **3270.51** | **44** | **29** | **-21** |
| **R superior frontal gyrus, medial segment** | **6** | **71** | **3117.84** | **3** | **32** | **41** |
| **R parahippocampal gyrus** |  | **23** | **3094.36** | **16** | **-14** | **-27** |
| **R cerebellum** |  | **299** | **3024.60** | **25** | **-40** | **-42** |
| R cerebellum |  |  | 2913.04 | 21 | -38 | -49 |
| **R cerebellum** |  | **3127** | **3003.19** | **24** | **-67** | **-60** |
| R cerebellum |  |  | 2978.87 | 39 | -53 | -61 |
| R cerebellum |  |  | 2968.16 | 33 | -59 | -64 |
| **R inferior frontal gyrus, orbital part** | 11 | **1343** | **2968.60** | **36** | **48** | **-20** |
| R inferior frontal gyrus, orbital part | 11 |  | 2963.51 | 36 | 48 | -11 |
| R inferior frontal gyrus, orbital part | 11 |  | 2962.13 | 27 | 35 | -20 |
| **L cerebellum** |  | **1** | **2903.46** | **-15** | **-31** | **-23** |
| **R lingual gyrus** | **18** | **1** | **2903.46** | **3** | **-74** | **-12** |
| **L superior temporal gyrus** | **41** | **8** | **2903.46** | **-50** | **-18** | **6** |
| **R cerebellum** |  | **566** | **2880.98** | **45** | **-61** | **-23** |
| R inferior temporal gyrus | 37 |  | 2867.57 | 51 | -56 | -25 |
| R inferior temporal gyrus | 37 |  | 2754.58 | 46 | -49 | -25 |
| **R middle frontal gyrus** | **6** | **379** | **2836.25** | **46** | **3** | **55** |
| R middle frontal gyrus | 8 |  | 2693.46 | 40 | 8 | 44 |
| **R cerebellum** |  | **10** | **2834.10** | **40** | **-44** | **-54** |
| **R cerebellum** |  | **12** | **2834.10** | **30** | **-41** | **-40** |
| **L cerebellar vermal lobules VI-VII** |  | **253** | **2776.50** | **-1** | **-77** | **-22** |
| L cerebellar vermal lobules VI-VII |  |  | 2720.55 | 2 | -71 | -17 |
| **R inferior occipital gyrus** | **19** | **80** | **2752.67** | **47** | **-78** | **-17** |
| **L inferior temporal gyrus** | **20** | **194** | **2740.53** | **-42** | **-5** | **-34** |
| L inferior temporal gyrus | 20 |  | 2726.95 | -36 | -10 | -39 |
| **R cerebellum** |  | **5** | **2712.51** | **35** | **-40** | **-39** |
| **L inferior temporal gyrus** | **20** | **3** | **2712.51** | **-46** | **-15** | **-26** |
| **L temporal pole** | **38** | **5** | **2712.51** | **-45** | **23** | **-26** |
| **R superior frontal gyrus, medial segment** | **9** | **2** | **2712.51** | **3** | **49** | **34** |

Cluster size = number of voxels in each significant cluster; TFCE = threshold free cluster enhancement, represents a statistical value, which combines voxel-height and cluster size; MNI coordinates (mm) = location of the maximum intensity voxel, given as spatial coordinate values in standard Montreal Neurological Institute coordinate space (mm). All the areas were significant at *p*<0.05, FWE corrected at a TFCE-based result.

***Table S6. Gray matter volume loss associated with volume of the left cholinergic cell group Ch4 in AD.***

| **Anatomical region** | **BA** | **Cluster size** | **TFCE** | **MNI coordinates** | | |
| --- | --- | --- | --- | --- | --- | --- |
|  |  |  |  | **x** | **y** | **z** |
| **L putamen** |  | **270612** | **28539.21** | **-22** | **-1** | **-12** |
| R amygdala |  |  | 25745.45 | 21 | 2 | -15 |
| L anterior insula | 13 |  | 25258.06 | -34 | 8 | -15 |
| **L supplementary motor cortex** |  | **72** | **16540.16** | **-3** | **22** | **45** |
| **L middle frontal gyrus** |  | **1166** | **16139.76** | **-23** | **42** | **23** |
| L middle frontal gyrus |  |  | 16120.36 | -28 | 38 | 28 |
| L middle frontal gyrus |  |  | 13997.13 | -28 | 47 | 16 |
| **R superior frontal gyrus** | **6** | **846** | **15556.93** | **21** | **1** | **58** |
| R superior/middle frontal gyrus |  |  | 13264.65 | 27 | 11 | 56 |
| R superior frontal gyrus |  |  | 13196.09 | 20 | 16 | 53 |
| **L inferior frontal gyrus, triangular part** |  | **25** | **15546.95** | **-44** | **35** | **2** |
| **L caudate** |  | **107** | **14784.41** | **-17** | **6** | **12** |
| **L precentral gyrus** |  | **1342** | **14376.08** | **-52** | **-9** | **42** |
| L precentral/postcentral gyrus | **6** |  | 14207.45 | -57 | -7 | 34 |
| L precentral gyrus | **6** |  | 14083.25 | -45 | -8 | 31 |
| **R hippocampus** |  | **33** | **14284.40** | **20** | **-25** | **-7** |
| **R posterior insula** |  | **17** | **14015.34** | **45** | **-4** | **1** |
| **R planum polare** |  | **68** | **14005.16** | **52** | **0** | **-2** |
| **R precuneus** | **5** | **328** | **13985.42** | **8** | **-43** | **54** |
| R posterior cingulate gyrus |  |  | 13253.05 | 12 | -38 | 38 |
| **R precuneus** |  | **12** | **13257.81** | **14** | **-56** | **45** |
| **brain stem** |  | **97** | **13170.92** | **-1** | **-37** | **-14** |
| **R hippocampus** |  | **5** | **13159.95** | **25** | **-24** | **-11** |
| **L hippocampus** |  | **3** | **13159.95** | **-35** | **-32** | **-10** |
| **L superior frontal gyrus, medial segment** |  | **3** | **13159.95** | **-19** | **56** | **-4** |
| **R caudate** |  | **74** | **13159.95** | **15** | **8** | **11** |
| R caudate |  |  | 12320.06 | 14 | 15 | 7 |
| **R middle frontal gyrus** |  | **3227** | **13142.99** | **40** | **22** | **29** |
| R middle frontal gyrus | 9 |  | 12804.47 | 44 | 20 | 38 |
| R middle frontal gyrus |  |  | 12733.23 | 41 | 10 | 33 |
| **R supplementary motor cortex** | **6** | **7** | **12324.51** | **3** | **4** | **65** |
| **R superior frontal gyrus** |  | **12** | **12322.63** | **11** | **28** | **47** |
| **L inferior frontal gyrus, opercular part** | **44** | **6** | **12321.54** | **-45** | **12** | **9** |
| **L hippocampus** |  | **1** | **12320.06** | **-37** | **-29** | **-13** |
| **L hippocampus** |  | **1** | **12320.06** | **-36** | **-30** | **-12** |
| **R hippocampus** |  | **2** | **12320.06** | **37** | **-26** | **-10** |
| **R posterior insula** |  | **1** | **12320.06** | **38** | **-20** | **-5** |
| **L supramarginal gyrus** |  | **3** | **12320.06** | **-39** | **-40** | **36** |
| **R precuneus** | **5** | **3** | **12320.06** | **10** | **-45** | **57** |
| **L postecentral gyrus** |  | **1** | **12320.06** | **-37** | **-26** | **61** |
| **L superior frontal gyrus, medial segment** |  | **261** | **11628.19** | **11** | **62** | **14** |
| **R fusiform gyrus** | **20** | **331** | **11608.92** | **29** | **-36** | **-24** |
| R fusiform gyrus |  |  | 11557.09 | 28 | -36 | -15 |
| R fusiform gyrus | **35** |  | 11533.93 | 30 | -28 | -24 |
| **L middle frontal gyrus** |  | **163** | **11581.69** | **-37** | **45** | **5** |
| **L middle frontal gyrus** |  | **93** | **11529.72** | **-39** | **47** | **17** |
| **R middle frontal gyrus** |  | **14** | **11520.18** | **25** | **44** | **22** |
| **L superior frontal gyrus** | **6** | **22** | **11518.37** | **-16** | **13** | **57** |
| **Left precentral gyrus** |  | **3** | **11516.51** | **-51** | **1** | **22** |

Cluster size = number of voxels in each significant cluster; TFCE = threshold free cluster enhancement, represents a statistical value, which combines voxel-height and cluster size; MNI coordinates (mm) = location of the maximum intensity voxel, given as spatial coordinate values in standard Montreal Neurological Institute coordinate space (mm). All the areas were significant at *p*<0.001, FWE corrected at a TFCE-based result.

***Table S7. Gray matter volume loss associated with volume of the right cholinergic cell group Ch4 in AD.***

| **Anatomical region** | **BA** | **Cluster size** | **TFCE** | **MNI coordinates** | | |
| --- | --- | --- | --- | --- | --- | --- |
|  |  |  |  | **x** | **y** | **z** |
| **R amygdala** | 34 | 70978 | 28814.20 | 20 | 0 | -14 |
| R entorhinal area | 34 |  | 20224.36 | 26 | 5 | -19 |
| R hippocampus |  |  | 20124.19 | 33 | -8 | -15 |
| **L amygdala** |  | 32851 | 17232.14 | -20 | -3 | -12 |
| L temporal pole |  |  | 17008.09 | -27 | 9 | -43 |
| L temporal pole | 38 |  | 16780.25 | -36 | 20 | -34 |
| **R postcentral gyrus** |  | 607 | 14485.10 | 47 | -18 | 36 |
| **R precentral/postcentral gyrus** |  |  | 13819.63 | 44 | -7 | 31 |
| **R precentral/postcentral gyrus** | **6** |  | 12652.30 | 50 | -4 | 26 |
| **L central operculum** |  | 2150 | 12796.72 | -38 | -15 | 18 |
| **L central operculum** | 13 |  | 12296.89 | -35 | -7 | 18 |
| **L central operculum** |  |  | 12204.17 | -58 | -11 | 16 |
| **L thalamus** |  | 646 | 12784.08 | -6 | -15 | -3 |
| **L thalamus** |  |  | 12652.30 | -9 | -18 | 4 |
| **L thalamus** |  |  | 12652.30 | -1 | -15 | 4 |
| **L hippocampus** |  | 7 | 12652.30 | -38 | -19 | -18 |
| **R hippocampus** |  | 7 | 12652.30 | 37 | -26 | -10 |
| **L supplementary motor gyrus** |  | 5532 | 12618.89 | -8 | 1 | 47 |
| **L supplementary motor gyrus** | 24 |  | 12600.10 | -7 | -7 | 45 |
| **L middle cingulate gyrus** | 31 |  | 12266.44 | -7 | -15 | 47 |
| **L thalamus** |  | 1721 | 12369.50 | -17 | -34 | 1 |
| **L thalamus** |  |  | 12197.20 | -18 | -29 | -8 |
| **L thalamus** |  |  | 11889.76 | -22 | -29 | 9 |
| **R middle frontal gyrus** |  | 414 | 12197.94 | 33 | 51 | 1 |
| **R superior frontal gyrus** |  |  | 11958.92 | 24 | 55 | -1 |
| **L planum temporale** | 41 | 28 | 12147.84 | -57 | -22 | 9 |
| **L middle frontal gyrus** |  | 481 | 12085.96 | -44 | 23 | 17 |
| **L inferior frontal gyrus, triangular part** |  |  | 11978.33 | -42 | 31 | 11 |
| **L middle frontal gyrus** |  |  | 11911.61 | -48 | 22 | 24 |
| **R parietal operculum** |  | 197 | 12020.44 | 53 | -23 | 30 |
| **R supramarginal gyrus** |  |  | 11930.78 | 54 | -23 | 38 |
| **R frontal pole** |  | 30 | 11953.80 | 12 | 58 | -11 |
| **R medial orbital gyrus** |  | 19 | 11927.45 | -13 | 44 | -18 |
| **L fusiform gyrus** |  | 1 | 11911.61 | -26 | -4 | -42 |
| **L fusiform gyrus** |  | 4 | 11911.61 | -35 | -12 | -25 |
| **L fusiform gyrus** |  | 1 | 11911.61 | -42 | -21 | -20 |
| **R hippocampus** |  | 1 | 11911.61 | 29 | -22 | -9 |
| **L superior temporal gyrus** |  | 5 | 11911.61 | -51 | -16 | -4 |
| **R parietal operculum** |  | 11 | 11911.61 | 55 | -21 | 24 |
| **R parietal operculum** |  | 1 | 11911.61 | 50 | -23 | 26 |
| **L fusiform gyrus** |  | 457 | 11583.36 | -25 | -41 | -16 |
| **L fusiform gyrus** | **20** |  | 11513.85 | -38 | -31 | -22 |
| **L fusiform gyrus** | **36** |  | 11488.03 | -30 | -32 | -20 |
| **R supplementary motor cortex** | **8** | 44 | 11494.08 | 5 | 15 | 55 |
| **L caudate** |  | 10 | 11452.18 | -18 | 6 | 12 |
| **L superior frontal gyrus, medial segment** |  | 9 | 11446.37 | -6 | 27 | 31 |

Cluster size = number of voxels in each significant cluster; TFCE = threshold free cluster enhancement, represents a statistical value, which combines voxel-height and cluster size; MNI coordinates (mm) = location of the maximum intensity voxel, given as spatial coordinate values in standard Montreal Neurological Institute coordinate space (mm). All the areas were significant at *p*<0.001, FWE corrected at a TFCE-based result.

***Table S8.* Gray matter volume loss associated with volume of the left cholinergic cell group Ch4 in controls.**

| **Anatomical region** | **BA** | **Cluster size** | **TFCE** | **MNI coordinates** | | |
| --- | --- | --- | --- | --- | --- | --- |
|  |  |  |  | **x** | **y** | **z** |
| **L amygdala** |  | **64417** | **26840.28** | **-20** | **-1** | **-13** |
| L ventral diencephalon |  |  | 19924.83 | -10 | 1 | -11 |
| R amygdala |  |  | 17607.97 | 22 | -2 | -12 |
| **L cerebellar vermal lobule VIII** |  | **2124** | **12277.12** | **-7** | **-63** | **-37** |
| L cerebellum, lobule IX |  |  | 12119.58 | -12 | -57 | -39 |
| L cerebellar vermal lobule X |  |  | 12036.95 | 0 | -49 | -28 |
| **R/L cerebellum, lobule IX** |  | **1246** | **12179.81** | **1** | **-47** | **-49** |
| R cerebellum, lobule IX |  |  | 12175.28 | 8 | -44 | -53 |
| R cerebellum, lobule IX |  |  | 12133.74 | 15 | -41 | -48 |
| **R cerebellum, lobule VIII-IX** |  | **233** | **11951.25** | **12** | **-58** | **-39** |
| R cerebellum, lobule IX |  |  | 11951.25 | 9 | -55 | -32 |
| R cerebellum, lobule VIII |  |  | 10770.77 | 10 | -64 | -34 |
| **L cerebellum, lobule IX** |  | **440** | **11795.72** | **-15** | **-45** | **-47** |
| L cerebellum, lobule VIII |  |  | 11311.23 | -24 | -39 | -47 |
| **R cerebellum, lobule VI** |  | **82** | **11765.59** | **9** | **-61** | **-28** |
| **L middle temporal gyrus** | **21** | **2877** | **11731.89** | **-65** | **-28** | **-4** |
| L middle temporal gyrus | 21 |  | 11709.16 | -64 | -34 | -15 |
| L middle temporal gyrus | 21 |  | 11425.74 | -64 | -44 | -8 |
| **L precentral gyrus** | **6** | **2949** | **11624.49** | **-59** | **-2** | **21** |
| L precentral gyrus | 6 |  | 11576.20 | -59 | -4 | 29 |
| L postcentral gyrus | 43 |  | 11382.21 | -59 | -10 | 18 |
| **R inferior frontal gyrus, orbital part** | **47** | **1070** | **11212.70** | **38** | **22** | **-17** |
| **R parahippocampal gyrus** |  | **3** | **10770.77** | **19** | **-27** | **-20** |
| **L thalamus** |  | **1** | **10770.77** | **-6** | **0** | **2** |
| **L superior temporal gyrus** | **22** | **169** | **10742.30** | **-63** | **-48** | **15** |
| **L fusiform gyrus** | **19** | **1254** | **10661.14** | **-30** | **-63** | **-16** |
| L cerebellum exterior |  |  | 10619.19 | -36 | -65 | -22 |
| L fusiform gyrus | 19 |  | 10597.33 | -24 | -70 | -14 |
| **R superior frontal gyrus, medial segment** | **10** | **316** | **10512.06** | **2** | **46** | **15** |
| R superior frontal gyrus, medial segment | 9 |  | 10492.33 | 11 | 54 | 19 |
| **L postcentral gyrus** | **4** | **254** | **10507.58** | **-33** | **-32** | **61** |
| L precentral gyrus | 4 |  | 10501.77 | -41 | -26 | 63 |
| L postcentral gyrus | 3 |  | 10379.13 | -32 | -32 | 70 |
| **L postcentral gyrus** | **3** | **23** | **10448.20** | **-45** | **-20** | **58** |
| **L superior frontal gyrus, medial segment** | **10** | **1** | **10394.32** | **-1** | **48** | **15** |
| **R superior frontal gyrus, medial segment** | **10** | **4** | **10377.06** | **11** | **51** | **28** |

Cluster size = number of voxels in each significant cluster; TFCE = threshold free cluster enhancement, represents a statistical value, which combines voxel-height and cluster size; MNI coordinates (mm) = location of the maximum intensity voxel, given as spatial coordinate values in standard Montreal Neurological Institute coordinate space (mm). All the areas were significant at *p*<0.001, FWE corrected at a TFCE-based result.

***Table S9. Gray matter volume loss associated with volume of the right cholinergic cell group Ch4 in controls.***

| **Anatomical region** | **BA** | **Cluster size** | **TFCE** | **MNI coordinates** | | |
| --- | --- | --- | --- | --- | --- | --- |
|  |  |  |  | **x** | **y** | **z** |
| **R amygdala** |  | **68782** | **42401.70** | **21** | **-2** | **-12** |
| R hippocampus |  |  | 27126.62 | 15 | -11 | -15 |
| L amygdala |  |  | 26771.45 | -20 | -3 | -12 |
| **L thalamus** |  | **1525** | **17532.74** | **-22** | **-31** | **4** |
| L thalamus |  |  | 17417.48 | -23 | -31 | -4 |
| L thalamus |  |  | 17417.48 | -16 | -36 | 1 |
| **L caudate** |  | **66** | **16965.12** | **-16** | **5** | **8** |
| **R caudate** |  | **17** | **16965.12** | **20** | **-9** | **20** |
| **R cerebellum, lobule IX** |  | **470** | **16342.22** | **15** | **-41** | **-45** |
| **L temporal pole** | **38** | **54** | **16055.46** | **-27** | **16** | **-32** |
| **L cerebellum, lobule IX** |  | **650** | **16051.46** | **-3** | **-46** | **-44** |
| L cerebellum, lobule IX |  |  | 16021.09 | -11 | -47 | -44 |
| R cerebellum, lobule IX |  |  | 15650.42 | 4 | -46 | -38 |
| **L caudate** |  | **2** | **16033.56** | **-17** | **0** | **16** |
| **L cerebellar vermal lobules VIII-X** |  | **296** | **15930.44** | **-6** | **-59** | **-31** |
| **L middle frontal gyrus** | **10** | **999** | **15408.94** | **-26** | **49** | **13** |
| L middle frontal gyrus | 10 |  | 15353.08 | -37 | 47 | 10 |
| L superior frontal gyrus | 10 |  | 15280.37 | -20 | 53 | 6 |
| **L inferior frontal gyrus, orbital part** | **10** | **1290** | **15394.24** | **-40** | **49** | **-7** |
| L inferior frontal gyrus, orbital part | 47 |  | 15380.49 | -38 | 41 | -10 |
| L triangular part of the inferior frontal gyrus | 47 |  | 15374.99 | -43 | 32 | 0 |
| **R temporal pole** | **38** | **679** | **15365.36** | **52** | **10** | **-25** |
| R temporal pole | 38 |  | 15330.87 | 44 | 11 | -28 |
| R temporal pole | 38 |  | 15183.15 | 36 | 13 | -27 |
| **L inferior frontal gyrus, orbital part** | **47** | **184** | **15175.87** | **-35** | **32** | **-15** |
| L inferior frontal gyrus, orbital part | 47 |  | 15122.13 | -37 | 40 | -15 |
| **R inferior frontal gyrus, orbital part** | **47** | **192** | **15142.02** | **38** | **23** | **-22** |
| R inferior frontal gyrus | 47 |  | 15138.01 | 31 | 20 | -19 |
| R inferior frontal gyrus, orbital part | 47 |  | 15123.84 | 45 | 25 | -17 |
| **R middle temporal gyrus** | **21** | **29** | **15138.29** | **51** | **2** | **-30** |
| **R inferior temporal gyrus** | **21** | **20** | **15131.54** | **44** | **-3** | **-38** |

Cluster size = number of voxels in each significant cluster; TFCE = threshold free cluster enhancement, represents a statistical value, which combines voxel-height and cluster size; MNI coordinates (mm) = location of the maximum intensity voxel, given as spatial coordinate values in standard Montreal Neurological Institute coordinate space (mm). All the areas were significant at *p*<0.001, FWE corrected at a TFCE-based result.
